# RNA virus-mediated changes in organismal oxygen consumption rate in young and old *Drosophila melanogaster* males

**DOI:** 10.18632/aging.204593

**Published:** 2023-03-22

**Authors:** Eli Hagedorn, Dean Bunnell, Beate Henschel, Daniel L. Smith, Stephanie Dickinson, Andrew W. Brown, Maria De Luca, Ashley N. Turner, Stanislava Chtarbanova

**Affiliations:** 1 Department of Biological Sciences, University of Alabama, Tuscaloosa, AL 35401, USA; 2 Present Address: Indiana University School of Medicine-Indianapolis, Medical Scientist Training Program, Indianapolis, IN 46202, USA; 3 Department of Epidemiology and Biostatistics, Indiana University School of Public Health-Bloomington, Biostatistics Consulting Center, Bloomington, IN 47405, USA; 4 Department of Nutrition Sciences, University of Alabama at Birmingham, Birmingham, AL 35233, USA; 5 Nathan Shock Center of Excellence in the Basic Biology of Aging, University of Alabama at Birmingham, Birmingham, AL 35294, USA; 6 Department of Applied Health Sciences, Indiana University, School of Public Health-Bloomington, Bloomington, IN 47405, USA; 7 Present Address: University of Arkansas for Medical Sciences and Arkansas Children’s Research Institute, Little Rock, AR 72202, USA; 8 Department of Biology, Jacksonville State University, Jacksonville, AL 36265, USA; 9 Center for Convergent Bioscience and Medicine, University of Alabama, Tuscaloosa, AL 35401, USA; 10 Alabama Life Research Institute, University of Alabama, Tuscaloosa, AL 35401, USA

**Keywords:** *Drosophila melanogaster*, aging, virus infection, single-fly respirometry, metabolism

## Abstract

Aging is accompanied by increased susceptibility to infections including with viral pathogens resulting in higher morbidity and mortality among the elderly. Significant changes in host metabolism can take place following virus infection. Efficient immune responses are energetically costly, and viruses divert host molecular resources to promote their own replication. Virus-induced metabolic reprogramming could impact infection outcomes, however, how this is affected by aging and impacts organismal survival remains poorly understood. RNA virus infection of *Drosophila melanogaster* with Flock House virus (FHV) is an effective model to study antiviral responses with age, where older flies die faster than younger flies due to impaired disease tolerance. Using this aged host-virus model, we conducted longitudinal, single-fly respirometry studies to determine if metabolism impacts infection outcomes. Analysis using linear mixed models on Oxygen Consumption Rate (OCR) following the first 72-hours post-infection showed that FHV modulates respiration, but age has no significant effect on OCR. However, the longitudinal assessment revealed that OCR in young flies progressively and significantly decreases, while OCR in aged flies remains constant throughout the three days of the experiment. Furthermore, we found that the OCR signature at 24-hours varied in response to both experimental treatment and survival status. FHV-injected flies that died prior to 48- or 72-hours measurements had a lower OCR compared to survivors at 48-hours. Our findings suggest the host’s metabolic profile could influence the outcome of viral infections.

## INTRODUCTION

Viral infections pose serious threats to the world’s population and are of particular concern to elderly individuals who are at an increased risk of death [1–3]. Viruses are obligate intracellular parasites that take over host cells and use the cellular machinery to replicate their genetic material and spread [4]. In the course of infection, viruses can hijack cellular energy and induce metabolic reprogramming in host cells to ensure sufficient availability of biomolecules, such as nucleotides and amino acids necessary for replication and for the formation of new virus particles [5, 6]. To counteract the infectious challenge and survive infection, multicellular hosts generally rely on two strategies, both considered components of immunity: resistance and tolerance [7, 8]. Resistance mechanisms coordinated as part of both innate and adaptive immunity detect, contain, and eliminate pathogenic microorganisms, while disease tolerance mechanisms reduce the immunopathological stress and tissue damage resulting from the infection at particular pathogen titer [8]. Mounting immune responses is energetically costly to the host and is associated with metabolic changes and the relocation of nutrients to support both immune activation and function, as well as tissue healing and repair [9–12]. Interestingly, however, how these processes are coordinated to promote survival of an infected aged host is not well understood. Therefore, investigating a host’s metabolic profile after viral infection could characterize physiological conditions favoring the host’s survival and potentially identify targets for novel antiviral treatments.

*D. melanogaster* represents a prime genetic model to decipher the cellular and molecular mechanisms of the activation of innate immunity, including antiviral defenses [11]. Because of its amenability for aging studies [13], this organism is also an excellent model in which to investigate age-dependent immune responses [14]. Flies are devoid of adaptive immunity, and solely rely on innate defenses. The major antiviral mechanism in *D. melanogaster* is RNA interference (RNAi), and more specifically the activation of the small interfering RNA (siRNA) pathway (reviewed in [15]). Additional antiviral defense mechanisms include plasmatocyte (macrophage-like cells in *Drosophila*)-mediated phagocytosis, apoptosis, and complex transcriptional responses (reviewed in [16, 17]). As is the case for vertebrate hosts [8], disease tolerance mechanisms in the context of viral infections are generally less well characterized, with one study implicating the H3 lysine 9 (H3K9) methyltransferase G9-alpha, an epigenetic regulator that plays a role in tolerance to infection with RNA viruses [18].

We previously used pathogenic infection of *D. melanogaster* with the Flock House virus (FHV) to investigate the changes in antiviral innate immune responses of aged hosts [19]. FHV is an *Alphanodavirus* that contains a bipartite, positive-sense, single-stranded RNA genome [20]. In infected insect cells, FHV replicates on the outer mitochondrial membrane inducing characteristic membrane invaginations known as replication spherules [21–23]. We showed that FHV-infected older flies died significantly sooner of FHV infection in comparison to younger flies, without accumulating higher virus loads [19]. This indicates that a mechanism of disease tolerance - rather than pathogen resistance - becomes impaired with age. Older flies also displayed a distinct transcriptional response to FHV, which is characterized by regulation of more genes and genes regulated to a greater extent in comparison to younger flies. Among these genes, stronger regulation of genes whose products function in biological processes such as metabolic processes, mitochondrial structure, and mitochondrial respiration is observed in older flies [19]. Mitochondria are central to cellular energy metabolism and deregulation of host’s bioenergetics could lead to altered metabolic function that impairs the ability to survive infection.

Although mitochondrial function, content, and morphology can be readily assayed in *D. melanogaster* using well-established protocols, this requires fly dissection or homogenization [24–26]. Therefore, such techniques involve the destruction of the sample at a unique timepoint making it impossible to conduct longitudinal assessments of metabolism in the same individual. Organismal metabolic rates depend on the amount of oxygen that is required for adenosine 5’-triphosphate (ATP) production and can be indirectly measured by the oxygen consumption rate (OCR). Because most of the body’s oxygen consumption occurs within mitochondria [27], OCR could provide estimates of nutrient utilization and indirectly, mitochondrial function. In this regard, in mammalian models, energy-conserving hypometabolism promotes disease tolerance in response to infection with the intracellular bacterium *Listeria monocytogenes* [28]. Furthermore, in *D. melanogaster*, infection with the *Drosophila* C virus (DCV), an RNA virus of positive strand, leads to metabolic rate depression (a term referring to the downregulation of organismal metabolic rate leading to energy saving) [12]. However, whether this change is also part of a disease tolerance mechanism is not known.

Here, we hypothesized that the *Drosophila* metabolic rate is deregulated following FHV infection in an age-dependent fashion. To test this hypothesis, we performed longitudinal, whole-organism respirometry at 24-, 48-, and 72-hours post-treatment in young and old male flies. Respirometry was performed using the Loligo Systems Microplate, which allows OCR measurements of 20 individual flies at a time. Individual survival status prior to each measurement was also recorded. This allowed us to also investigate whether changes in OCR influence infection survival.

Our results show that FHV infection significantly reduces organismal OCR compared to Tris-injected controls; however, we did not observe a significant change in OCR with aging. Interestingly, flies that died prior to 48-hours post-treatment measurements exhibited a significantly lower OCR at 24 h post-treatment compared to survivors. These findings suggest that the host’s metabolic profile could influence the outcome of viral infections.

## RESULTS

### FHV-infection alters organismal oxygen respiration, peaks at a certain time of day and is not specific to aged *Drosophila*

**Figure 1:**
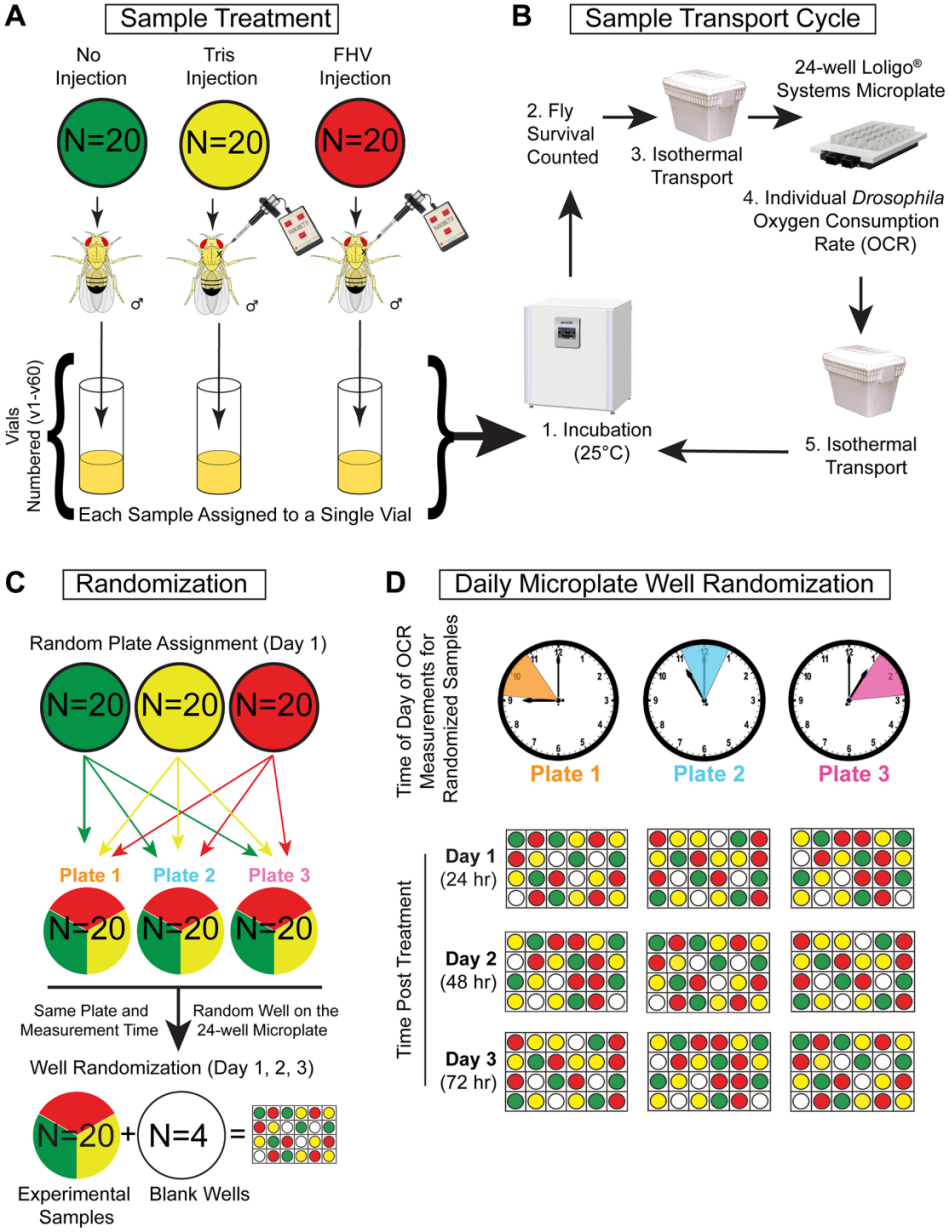
**Single-fly whole-organism respirometry protocol and sample randomization.** (**A**–**D**) Represented is the flow chart of treatment, handling, and randomization procedures to which each cohort of *n* = 60 flies were subjected during four separate weeks (2 young cohorts and 2 aged cohorts) for a total of 240 flies. (**A**) During each round of whole organism respirometry, *n* = 60 Young (4–8 days-old) or *n* = 60 Aged (28–32 days old) male *Oregon-R* flies were separated into 3 treatment groups of *n* = 20 flies and subjected to Non-injected (Ni), Tris-injection, or FHV-injection treatment. After treatment, each fly was placed into a single vial and placed in a 25°C incubator (12 h light:12 h dark cycle). (**B**) Prior to respiration measurement, flies were taken out from the 25°C incubator and checked for viability. Next, they were transported in an isothermal container to prevent variations in temperature. At the Comparative Energetics Organismal Core facility, a 24-well Microplate (Loligo Systems) was used to measure the oxygen consumption rate (OCR) of individual flies. After measurement, flies were placed back into respective vials and returned to the 25°C incubator in an isothermal container. The sample transport cycle was repeated for the duration of the longitudinal OCR measurements. (**C**) On each microplate, a cohort of *n* = 20 randomly selected flies were measured alongside *n* = 4 blank wells (negative controls). (**D**) On each subsequent timepoint (24 h, 48 h, and 72 h post-treatment), well position was re-randomized for each microplate cohort resulting in the cohort being measured at the same time each day.

The experimental design of the study is outlined in Figure 1. As shown in Table 1, we observed a significant main effect of ‘Treatment’ (*p* = 0.032), ‘Time Post-Treatment’ (*p* < 0.0001), and ‘Zeitgeber Time’ (*p* = 0.028) on OCR. However, contrary to our prediction, we did not find any effect of age at injection (‘Age’) on OCR (Table 1 and Supplementary Figure 1A).

**Table 1:** Repeated measures ANOVA of main and interaction effects on organismal oxygen consumption rate (OCR) of young and old *D. melanogaster* males subjected to non-injected, tris-injected of FHV-injected treatments.

**Effect**	**DF between groups**	**DF within groups**	**F value**	**Pr > F (*p*-value)**
*‘Well Position’*	23	312	1.54	0.055
*‘Zeitgeber Time’*	2	232	3.65	**0.028**
*‘Treatment’*	2	232	3.50	**0.032**
*‘Age”*	1	232	0.16	0.688
*‘Time Post-Treatment’*	2	316	11.14	**<0.0001**
‘Treatment * Age’	2	232	0.48	0.617
‘Treatment * Time Post-Treatment’	4	316	0.70	0.590
‘Age * Time Post-Treatment’	2	316	4.70	**0.010**
‘Age * Treatment * Time Post-Treatment’	4	316	1.80	0.128

Abbreviation: DF: Degrees of freedom. Significant *p* values (*p* < 0.05) are bolded.

**Figure 2:**
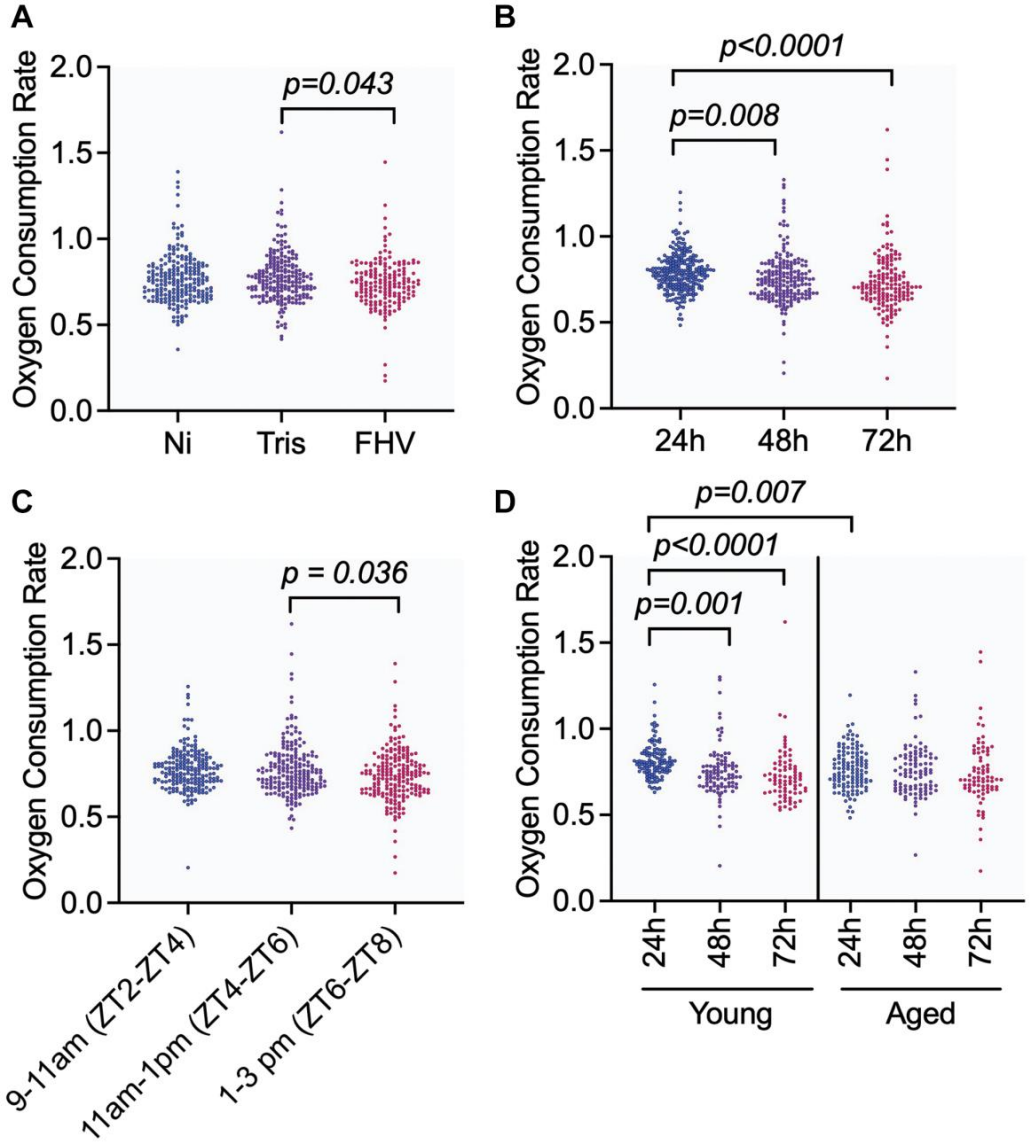
**Injection of *D. melanogaster* males with FHV decreases levels of organismal OCR in both young and aged flies, but OCR is also influenced by the time post-treatment in young flies and the time of the day during which measurements are performed.** (**A**–**D**) Graphs showing pairwise comparisons of variables with significant main effect on OCR: ‘Treatment’ (**A**), ‘Time Post-Treatment’ (**B**), and ‘Zeitgeber Time’ (**C**) and interaction effect: ‘Age * Time Post-Treatment’ (**D**). OCR measurement values for individual datapoints are plotted. Significant comparisons were determined by post hoc Tukey-Kramer tests within each panel as described in methods. Each symbol represents an individual OCR measurement on a fly.

Follow-up pairwise comparisons were used to determine whether significant differences in OCR were observed between specific levels of ‘Treatment’, ‘Time Post-Treatment’, and ‘Zeitgeber Time’. OCR of FHV-injected cohorts was significantly reduced compared to Tris- injected controls (*p* adj = 0.043). The OCR of Tris- and Ni flies were not significantly different from each other (*p* adj = 0.974), which indicates that changes in OCR are not associated with the injury induced by the nano-injection protocol (Figure 2A). Although Ni and FHV OCR measurements were not statistically significantly different, the difference was consistent with a downward trend (*p* adj = 0.068 between Ni and FHV cohorts) (Figure 2A). For OCR measurements at different times post-treatment, we observed that OCR measurements at 24 h were significantly higher compared to measurements at 48 h (*p* adj = 0.008) and at 72 h (*p* < 0.0001) (Figure 2B).

Zeitgeber Time (ZT) is a standardized unit of time based on an environmental variable such as the 12 h:12 h light:dark cycle, where ZT0 corresponds to the beginning of day (light phase), and ZT12 corresponds to the beginning of night (dark phase) [29]. In our study, Zeitgeber Time 0 (ZT0) was set at 7 am CST. OCR during the 1–3 pm interval (ZT6-8) were significantly different relative to the 11am-1pm interval (ZT4-6; *p* adj = 0.036) (Figure 2C). There was no significant difference in OCR between the 9–11 am (ZT2-4) and the 11 am–1 pm (ZT4-6) intervals (*p* adj = 0.948) nor between 9–11 am (ZT2-4) and the 1–3 pm (ZT6-8) intervals (*p* adj = 0.082). Altogether, our results indicate that lower levels of oxygen respiration are specifically associated with FHV infection in both young (4–8 days-old) and aged (28–32 days-old) flies in comparison to Tris-injected controls, and that OCR significantly changes during the experiment. The time of day during which measurements are conducted influences OCR, reinforcing the need to account for this variable by experimentally controlling for time of day, such as by stratifying randomization of treatment within time of day.

### Respiration in younger flies significantly decreases during the experiment, while respiration in older flies remains constant throughout the experiment

Our statistical analysis also showed a significant interaction between age at injection and OCR measurements at different times post-treatment (24 h, 48 h, or 72 h) (‘Age * Time Post-Treatment’ (*p* = 0.010). However, there was no significant effect of ‘Age * Treatment’, ‘Treatment * Time Post-Treatment’, and ‘Age * Treatment * Time Post-Treatment’ interactions on OCR (Table 1 and Supplementary Figure 1B–1D).

Pairwise comparisons of OCR measurements at different times post-treatment stratified by young and aged cohorts showed that the OCR of young flies significantly decreased from 24 h to 72 h post-treatment (24 h–48 h: *p* adj = 0.001, 24 h–72 h: *p* adj < 0.0001, Figure 2D). In contrast, the respiration observed in aged flies did not significantly vary at the three different experimental timepoints (24 h–48 h: *p* adj = 0.792, 24 h–72 h: *p* adj = 0.486, Figure 2D). Comparing age of flies within time post-treatment, we observed a significant difference in the measured OCR between aged and young flies at the 24 h timepoint (*p* adj = 0.007) (Figure 2D). None of the other pairwise comparisons within the ‘Age * Time post-treatment’ interaction effect were significant (48 h: *p* adj = 0.903 and 72 h: *p* adj = 0.199). OCR levels in response to all combinations of treatments by time post-treatment and by age are shown in Supplementary Figure 1B–1D. We note that older flies maintain more constant OCR following different treatments than younger flies. These results suggest that young flies can modulate their OCR in response to experimental treatments, including FHV infection, while this response in older flies remains constant.

### Respirometry experimental conditions increase *Drosophila* mortality, particularly in FHV-injected flies

During the process of measuring OCR, we recorded the survival status of each fly (alive or dead) prior to measurements at each post-treatment timepoint. This allowed us to compare post-treatment mortality between treatments within post-treatment time using Chi-square (χ^2^) tests.

**Figure 3:**
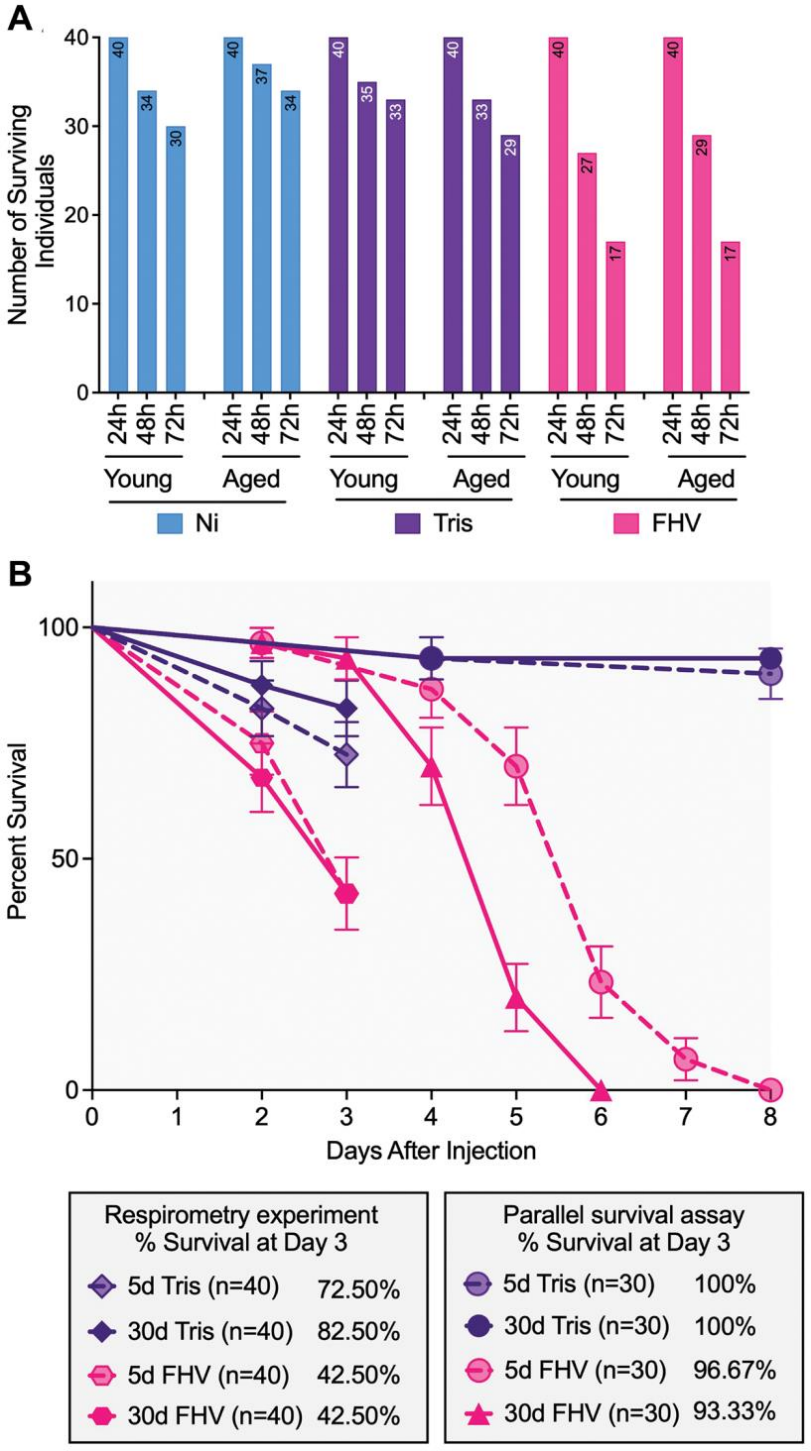
**FHV-injected cohorts display higher mortality compared to controls throughout the experiment, and mortality of young and aged cohorts is comparable among treatment groups.** (**A**) Bar graphs showing the distribution of individual survival within each ‘Treatment’ group at 24 h, 48 h and 72 h post-treatment in both young and old flies (*n* = 40 starting in each age-by-treatment group). (**B**) Survival curves comparing mortality of Tris- and FHV-injected flies recorded during the respirometry experiment (*n* = 40 flies per experimental condition) and during an independent survival assay carried in parallel (*n* = 30 flies per experimental condition). A significant difference between 5- and 30-days-old flies is observed following FHV infection (*p* < 0.0001), but not Tris injection (*p* = 0.648) in the parallel survival assay, based on a log-rank test. At 3 days post-treatment, no significant difference is observed between young and aged FHV-infected flies in both the respirometry experiment (*p* = 0.827) and the parallel survival assay (*p* = 0.562) based on a log-rank test.

As shown in Figure 3A, prior to the 24 h measurements, no mortality was observed in any ‘Treatment’ group. However, at 48 h a total of 44 flies had died with significant differences by treatment (χ^2^_(2)_ = 9.07, *p* = 0.011): more FHV injected flies (29%) had died compared to Ni (11%) and Tris-injected controls (15%). Significant differences in survival by treatment were also observed at 72 h (χ^2^_(2)_ = 31.65, *p* < 0.001)). While a total of 80 flies had died, more FHV injected flies (57.5%) had died compared to Ni (20%) and Tris-injected controls (22.5%). Logistic regression analysis at both 48 h (*p* = 0.013) and 72 h (*p* < 0.0001) timepoints corroborated these results. No significant differences in mortality by ‘Age’ within each of the three treatments were found (48 h - Ni: *p* = 0.481, Tris: *p* = 0.531, FHV: *p* = 0.459; 72 h - Ni: *p* = 0.264, Tris: *p* = 0.284, FHV: *p* = 1.000). Similarly, logistic regression analysis indicated non-significant differences in mortality with age (48 h - Ni: *p* = 0.297, Tris: *p* = 0.533, FHV: *p* = 0.460; 72 h - Ni: *p* = 0.268, Tris: *p* = 0.288, FHV: *p* > 0.999).

Next, we carried out a survival assay at 25°C in parallel with the respirometry experiment. The rationale for the additional experiment was that in our previous work [19] all survival assays were done at a lower temperature (22°C), while the assessment of mortality in the course of the respirometry experiment herein was done at 25°C. We found that mortality of the young and aged cohorts is comparable in Tris-injected flies (Log-rank test *p* = 0.648), but does differ significantly in FHV-injected flies (Log-rank test *p* < 0.0001) (Figure 3B). However, at the first 72 h post-treatment in the survival experiment (consistent with the timeframe of the microplate measurements), we observed comparable survival between young (96.67% survival) and aged (93.33% survival) FHV-injected cohorts (log-rank test *p* = 0.562). Both young and aged Tris-injected cohorts displayed 100% survival at 72 h post-treatment (Figure 3B). When plotted on the same graph, the values of % flies surviving Tris or FHV infection from the respirometry experiment also showed no significant difference as a function of age (Tris: Log-rank test *p* = 0.295 and FHV: Log-rank test *p* = 0.827). We note, however, that the flies involved in the respirometry measurements experienced an accelerated mortality rate compared to the survival experiment (Tris Young: log-rank test *p* = 0.002, Tris Aged: log-rank test *p* = 0.017, FHV Young: log-rank test *p* < 0.0001 and FHV Aged: log-rank test *p* < 0.0001) (Figure 3B). These results suggest that the experimental conditions used in the respirometry experiment are associated with increased mortality.

### Lower OCR at 24 h post-treatment is associated with mortality after viral infection

**Figure 4:**
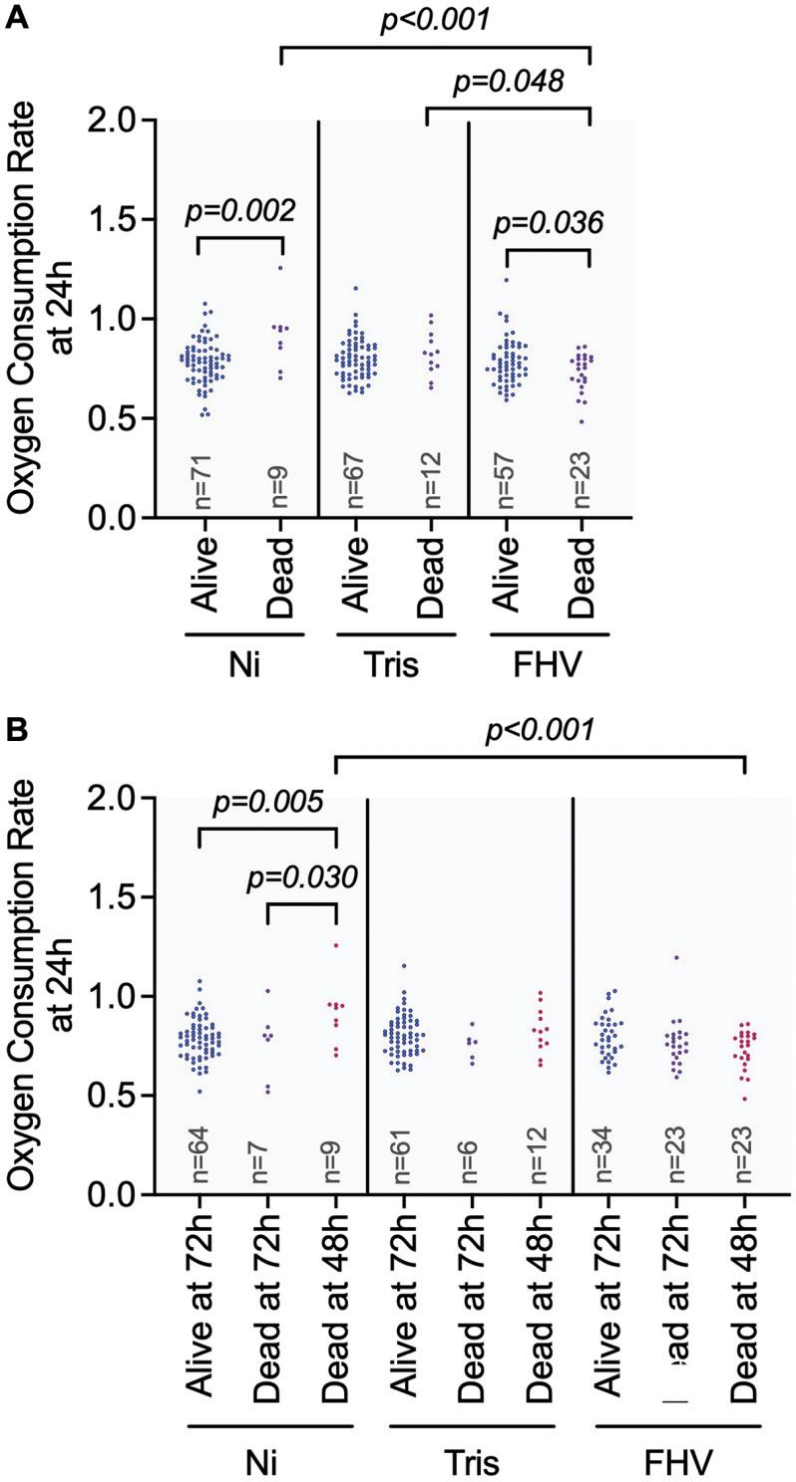
**Differences in OCR could influence the outcome of FHV infection.** (**A**, **B**) Graphs showing pairwise comparisons of variables with significant main effect on OCR at 24 h: ‘Treatment’ (**A**) and significant interaction effect on OCR: ‘Treatment * Survival 48- and 72-hours’ (**B**). OCR measurement values for individual datapoints are plotted. Significant comparisons were determined by post hoc Tukey-Kramer tests as described in methods. Each symbol represents an individual OCR measurement on a fly.

Type 3 ANCOVA analysis of the effects on OCR at 24 hours post-treatment (when all flies are living) shows that the main effect of ‘Treatment’ (*p* = 0.001) and the interaction effect of ‘Treatment * Survival Status at 48 h’ (*p* = 0.001) are significant effects that could explain variations in OCR at the 24 h timepoint (Table 2). We observed significant pairwise differences within treatment groups based on an individual fly’s survival status at 48 h (Figure 4A). The Ni flies that died prior to the 48 h measurements have a significantly higher OCR at 24 h (*p* adj = 0.002) relative to the flies that were alive at 48 h. In contrast, the FHV-injected flies that died prior to the 48 h measurements had a significantly lower OCR at 24 h (*p* adj = 0.036) when compared to the flies that were alive at 48 h. There was no significant difference in OCR at 24 h in Tris-injected flies with respect to their survival status at 48 h (*p* adj = 0.443). We observed significantly lower 24 h OCR for flies from the FHV-injected cohort that died prior the 48 h measurement in comparison to flies from the Ni (*p* adj < 0.001) and Tris-injected (*p* adj = 0.048) cohorts that died prior to the 48 h measurement. These results support the idea that the cause for the mortality observed at the 48 h post-treatment timepoint differs between the Ni- and FHV-injected groups with lower metabolic rates at the 24 h post-treatment timepoint.

**Table 2:** Type 3 ANCOVA of main and interaction effects on organismal oxygen consumption rate (OCR) at 24-hours post-treatment of young and old non-injected, tris-injected or FHV-injected *D. melanogaster* males that either survived or died at 48-hours post-treatment.

**Effect**	**DF between groups**	**DF within groups**	**F value**	**Pr > F (*p*-value)**
*‘Well position’*	23	232	1.07	0.386
*‘Zeitgeber Time’*	2	202	2.14	0.121
*‘Treatment’*	2	202	7.96	**0.001**
*‘Age”*	1	202	1.42	0.235
*‘Survival at 48-hours’*	1	202	3.00	0.085
‘Treatment * Survival at 48-hours’	2	202	7.40	**0.001**
‘Age * Treatment’	2	202	1.73	0.181
‘Age * Survival at 48-hours’	1	202	2.41	0.122
‘Age * Treatment * Survival at 48-hours’	2	202	1.04	0.356

Abbreviation: DF: Degrees of freedom. Significant *p* values (*p* < 0.05) are bolded.

### OCR at 24 hours is variable within treatment groups with respect to survival status at each timepoint

We next compared OCR at 24 h between flies that were dead before 48 h measurements, dead before 72 h measurements, and flies that survived the course of the experiment within their respective treatment groups (Figure 4B and Table 3). This was done to determine if an individual fly’s OCR provided any insight on their ability to survive stress, manipulation, injury, and/or viral infection.

**Table 3:** Type 3 ANCOVA of main and interaction effects on organismal oxygen consumption rate (OCR) at 24 hours post-treatment of young and old non-injected, tris-injected or FHV-injected *D. melanogaster* males that either survived or died at 48- and 72-hours post-treatment.

**Effect**	**DF between groups**	**DF within groups**	**F value**	**Pr > F (*p*-value)**
*‘Well position’*	23	196	0.98	0.497
*‘Zeitgeber Time’*	2	196	2.04	0.133
*‘Treatment’*	2	196	3.40	**0.035**
*‘Age”*	1	196	2.41	0.122
*‘Survival at 48- and 72-hours’*	2	196	2.58	0.078
‘Treatment * Survival 48- and 72-hours’	4	196	4.19	**0.003**
‘Age * Treatment’	2	196	2.15	0.119
‘Age * Survival at 48- and 72-hours’	2	196	1.08	0.342
‘Age * Treatment * Survival 48- and 72-hours	4	196	1.23	0.300

Abbreviation: DF: Degrees of freedom. Significant *p* values (*p* < 0.05) are bolded.

Similar to above, only the main effect for ‘Treatment’ (*p* = 0.035) and the interaction of ‘Treatment’ with the mortality indicator (*p* = 0.003) were significant. The Ni flies that died before 48 h had a significantly higher 24 h OCR compared to flies that were alive at 72 h (*p* adj = 0.005) and that died before 72 hours (*p* adj = 0.030). The Tris-injected flies did not have significant pairwise differences in their 24 h OCRs with respect to differences in survival status throughout the experiment (dead before 48 h compared to alive at 72 h: p adj = 0.806, dead before 72 h: *p* adj = 0.308). For FHV-injected flies that died before 48 h timepoint we found a lower 24 h OCR consistent with a downward trend (*p* adj = 0.056) than flies that were alive at 72 h. Yet, this interaction wasn’t significant. Additionally, there was no significant difference in OCR between FHV-injected flies that died before the 72 h measurement and the flies that survived the experiment (*p* adj = 0.530) (Figure 4B). Together, these results indicate that following FHV infection, lower OCR at 24 h is linked to mortality at the 48 h post-treatment timepoint.

## DISCUSSION

In this study, we investigated how infection with an RNA virus affects organismal metabolic rate as a function of age. We used male flies because our previous work characterizing the transcriptional changes following FHV infection with age was done in males, and we found older males to die faster of FHV infection than age-matched females [19]. Here, we utilized the Loligo Systems complete Microplate System to conduct longitudinal, whole-organism respirometry as a proxy of metabolic rate at a single fly resolution. It is worth noting that between the two controls in these experiments, we consider the Tris-injected cohort to be the more appropriate control when compared to the Non-injected cohort as it accounts for the injury sustained by injection. Additionally, we recorded the survival status of each fly prior to measurements at each post-treatment timepoint. With this approach, we were able to monitor both the OCR and mortality of each individual fly during three timepoints in the course of viral infection. Thus, this experiment assessed the interaction between post-treatment mortality, experimental variables (e.g., ‘Age’, ‘Treatment’, ‘Time Post-Treatment’), and changes in respiration quantified as OCR. To our knowledge, this is the first study that provides such measurements at the single-fly resolution using a *Drosophila*-virus interactions model in the context of aging and infection with an RNA virus.

### FHV affects organismal respiration and cellular metabolism

The significantly lower OCR observed in the FHV-injected cohort confirmed that FHV infection alters the host’s bioenergetics profile. On one hand, this likely represents virus-induced changes in organismal metabolism that correspond to mounting an efficient immune response by the host to fight the infection. On the other hand, this could represent the reprogramming of the cell by the virus to generate resources for virus replication and spread. However, in the present study, we did not confirm the hypothesis that metabolic rates differ significantly between young and aged cohorts following FHV infection. This hypothesis stemmed from our previous studies showing that 48 hours post FHV infection aged *Drosophila* downregulate the expression of more genes and to a greater extent, with function in the mitochondrial respiratory chain [19]. This suggests that even though oxidative phosphorylation (OXPHOS) could be impaired to a greater extent in older flies, it may not be the sole factor responsible for the increased mortality to FHV with age. Using OCR as a limited proxy for mitochondrial function assessment, we thus find that in comparison to the Tris-injected controls, FHV infection leads to decreased organismal OCR but does not present an overall age-specific effect. Of note is the fact that even though the average OCR was not significantly different between age groups, we observed a significant ‘Age * Time Post-Treatment’ interaction effect on OCR. Specifically, aged flies exhibited significantly lower OCR at 24 h in comparison with younger flies. This could reflect a possible lower metabolic rate due to an age-associated decline in mobility, which could possibly also explain the more robust changes in mitochondrial respiratory chain gene expression following FHV infection in older flies. Indeed, lower metabolic rates have previously been recorded for older *Drosophila* in comparison to younger flies [30, 31]. However, further studies are warranted in this system to confirm whether this is the case since OCR measurement cannot directly assess cellular respiration. Future analysis could employ higher resolution respirometry done on isolated mitochondria from young and aged cohorts to confirm whether any age-dependent changes in the electron transport chain function are detected after virus infection. Because this study was conducted in a time frame post FHV infection when comparable survival between age groups was expected, future experiments could be done to determine whether significant age-dependent changes in OCR take place at later timepoints when differences in survival between age groups are significant. This could reveal additional metabolic aspects of the response of the aged organism to viral infection.

Virus-specific metabolic changes occur not only to establish the appropriate bioenergetic state of the host, but to provide the appropriate metabolites necessary to generate infectious virions and promote virus spread [32]. For example, positive-strand RNA viruses utilize host cellular membranes to generate virus replication complexes for genome replication. Notably, FHV assembles its RNA replication complexes on mitochondrial outer membranes forming characteristic spherules [22, 23, 33]. These replication spherules could alter mitochondrial structure, which has been shown to impact mitochondrial function [34]. Published studies indicate that membrane lipid distribution and synthesis is important for functional replication complexes. Glycerophospholipid metabolism, specifically phosphatidylcholine biosynthesis, plays a critical role in FHV RNA replication. After FHV infection in cultured *Drosophila* S2 cells, several lipid metabolism genes are upregulated and associated with increased phosphatidylcholine accumulation. Interestingly, RNA interference-mediated downregulation of *Phosphocholine cytidylyltransferase Cct1* and *Cct2*, encoding essential enzymes in phosphatidylcholine synthesis, suppressed FHV RNA replication [35]. *Cct1* and *Cct2* are also significantly upregulated in both young and aged flies following FHV infection [19]. Together, our previous data and the results from the present study support the idea that following FHV infection a virally-induced metabolic shift occurs, a phenomenon known as the Warburg effect [36, 37]. The Warburg effect was originally characterized in cancer cells; however, many viruses alter their host’s metabolism in a similar fashion [38]. Some of the known hallmarks of this reprogramming include a reduction in the dependence of the cell on the mitochondrial electron transport chain and OXPHOS, and a preferential utilization of aerobic glycolysis leading to rapid ATP production and higher, lactate dehydrogenase (LDH)-dependent production of lactate [39]. LDH reversibly converts pyruvate to lactate, and is a key enzyme in the shift to aerobic glycolysis [40]. Although aerobic glycolysis is less efficient in generating ATP, it provides the cell with metabolic precursors, such as nucleotides, amino acids, and lipids that are necessary to construct virions to promote viral spread [32, 41]. Review of transcriptomic data from our previous study [19] confirmed that LDH was significantly upregulated within both young and aged, FHV-infected cohorts at 24 h and 48 h post infection, further supporting the idea that FHV infection induces the Warburg effect. Whether this is the case, and the exact implications of LDH activation in outcomes associated with virus infection could be addressed in future work. For instance, this could include measuring lactate levels as well as testing survival outcomes in young and aged cohorts of LDH mutants and comparing these outcomes with respective wild type controls. Furthermore, it would be of interest to determine whether FHV-triggered metabolic reprogramming in *Drosophila* also leads to other, non-metabolic effects that could be associated with the age-dependent mortality following virus infection. Studies in cancer cells have demonstrated that increased inflammation and accumulation of advanced glycation end products (AGEs) are among such effects [41]. Some AGEs have protein crosslinking or binding capabilities in the cell that could alter multiple aspects of protein homeostasis. These include blocking receptor binding, altering enzymatic activity or cleavage sites, and causing protein misfolding [41]. Future studies could address this question in *Drosophila*, and determine whether virus-induced metabolic reprogramming precedes the activation of innate immune pathways, as well as evaluate the age-dependency of these interactions.

### The longitudinal assessment of OCR reveals differences between age groups

One additional interesting finding in our study is that young flies showed progressive and significant decrease in OCR in the 48 h and 72 h after treatment, while OCR in aged flies remained constant throughout the three days of treatment.

Although during the experiment OCR decreased in both Ni and FHV-infected young flies, the result observed in young, FHV-infected flies is consistent with previous studies that looked at metabolic changes that take place in flies infected with another RNA virus, DCV [12]. In this study, the authors used open-flow CO_2_ respirometry on single flies to show that metabolic rate as measured by CO_2_ production decreases during the first four days of DCV infection. They proposed that the observed metabolic rate depression represents a response to the physiological stress resulting from DCV infection [12]. In mammals, decreased metabolism, or hypometabolism, is a survival strategy that promotes disease tolerance in response to infection with intracellular bacteria [28]. Older flies exhibit impaired disease tolerance to FHV [19], and here we show that metabolic rate depression does not occur in older flies in response to FHV in the first three days following treatment. It is therefore possible that as is the case in mammals, flies employ hypometabolism as a survival strategy that is part of a disease tolerance mechanism. It would be interesting in the future to test this hypothesis by comparing OCR in tolerance mutant flies such as the *G9a* mutants.

Recording the survival status of each individual prior to each measurement also allowed us to determine whether variations in OCR throughout the experiment influence an organism’s ability to survive different treatments. This study establishes host metabolism as a factor that influences the survival outcomes of viral infection, with a higher OCR at 24 h being correlated with longer survival in FHV-infected flies at the 48 h timepoint. This seems somewhat contradictory, as our OCR data suggests hypometabolism appears to be a survival strategy for young flies. FHV-injected flies that survived the experiment had significantly higher OCR than flies that died prior to the 48 h measurement timepoint. In contrast, Ni flies that survived to later timepoints had lower OCR at 24 h post-treatment. The Ni flies that died prior to the 48 h measurement experienced a significantly higher OCR at 24 h. We could speculate that Ni flies that experienced mortality could possess OXPHOS defects that trigger hypermetabolism. Indeed, a recent study in humans [42] has shown that hypermetabolism, characterized by increased volume of oxygen consumption (VO_2_) or OCR, is a phenotype associated with reduced lifespan. This suggests that FHV infection can induce a host metabolic response within the first 24 hours of infection that is distinct from experimental stressors. It is interesting that surviving, FHV-infected flies display a higher 24 h OCR, in contrast to the hypometabolic response seen in later timepoints. Previous studies using *D. melanogaster* infected with the Gram-negative bacterium *Providencia rettgeri* have shown two distinct phases of infection that correlate to the transition between pathogen resistance and disease tolerance [43]. The authors showed that this shift occurs following 72 hours of infection. It is possible that the shift from increased respiration in early timepoints to decreased respiration similarly reflects this shift in our study. Moreover, the fact that older flies do not undergo this shift towards hypometabolism may further explain the decreased disease tolerance observed in [19]. As the exact mechanisms by which hypometabolism promotes tolerance are not fully understood, *D. melanogaster* could serve as an excellent model to dissect the genetic and molecular bases of this process.

### Effect of non-viral factors on OCR and impact of OCR on survival

In the present study, we detected a significant effect of ‘Zeitgeber Time’ on OCR that has the potential to confound the other effects such as ‘Treatment’ that were significantly associated with OCR. However, prior to the experiment, a variation in OCR with respect to *Drosophila* circadian rhythm was expected [30, 44]. As a control for this variable, we randomly assigned flies of different treatments and ages to three microplates. Each microplate was measured at a specific daily interval across each timepoint post-treatment. Therefore, even though well position was randomized, each individual remained in the same plate and had their OCR measured at the same time each day. The randomization that we incorporated to our study also acted to control the effect of inter-well variance, since individual placement on the Loligo Systems Microplate was randomly chosen. Our results underscore the necessity of implementing experimental strategies to account for factors such as the time of day during which experiments are conducted.

The significant change in OCR as fly cohorts progressed through each post-treatment timepoint and the mortality observed during the experiment could have resulted from accumulating stressors during transport and during the oxygen measurement procedure. The latter involved manipulations such as taking in- and out- of the vial individual flies, as well as their placement and removal on and from the microplate, respectively. Although we cannot exclude that these parameters influenced OCR, we note that all experimental cohorts were subjected to the same protocols of transport and manipulation, thus accounting for their possible confounding effect on the study. Indeed, our results show a significant effect of ‘Treatment’ on OCR in FHV-infected cohorts, but not in Ni or Tris-injected flies.

The flies involved in our Loligo measurements experienced an accelerated mortality compared to the parallel survival assay, which did not involve transportation or manipulation required for OCR measurements. Thus, these factors can also potentially contribute to the accelerated mortality over the course of the respirometry experiment. Our goal was to conduct the respirometry analysis prior to the detectable differences in survival of FHV infection between young and aged flies. Based on our previous work showing that robust differences between young and aged FHV-infected cohorts exist at the level of their transcriptomes at timepoints before the mortality onset [19], we reasoned that similarly, we may be able to detect differences in metabolic rates between the age groups at comparable survival rates. The goal of the parallel survival assay was to confirm that in the first three days following Tris or FHV treatment, no significant age-dependent difference exists in survival of FHV infection. We note that in this assay, we did not use a randomization protocol to assign Tris or FHV treatment to the two age groups as we did in the respirometry experiment. Additionally, flies were not housed individually but rather in groups of 10 per vial. The parallel survival assay confirmed that at 25°C differences in mortality between age groups are not observable prior Day 4, therefore making it suitable to examine metabolic rates within the time frame of the first 72 h following the different experimental treatments. Despite the overall accelerated death in the respirometry experiment, possibly due to transport and manipulation, we note that similar to the parallel survival assay, there was no difference in mortality between age groups during the time frame of the experiment (e.g., 3 days post-treatment). We therefore concluded that OCR measurements can reproducibly be carried out in our host-virus interactions model revealing insights about virus-induced changes in host metabolism, and thus, making our system suitable for future studies.

Further studies are required to determine how FHV manipulates host metabolism and to identify factors specific to the metabolism of aged hosts that result in their increased susceptibility to infection. To further analyze the effects of FHV infection, whole-fly respirometry can be complemented by high-resolution respirometry measurements on isolated mitochondria. Additionally, genetic analysis of fly mutants for genes involved in metabolic reprogramming could provide further mechanistic insights about the role of this process in age-dependent responses and survival of virus infection. Combined with genetic analysis and whole-fly respirometry, isolated mitochondria assays will further elucidate the systemic effects of age and FHV infection upon *Drosophila* respiration and metabolic rates.

## MATERIALS AND METHODS

### *Drosophila* handling, aging and randomization protocols

Male *Oregon-R Drosophila melanogaster* were used in all experiments. *Oregon-R* stock (#2376) was obtained from Bloomington *Drosophila* Stock Center (Bloomington, IN, USA). Flies were maintained in vials containing Nutri-Fly Bloomington formulation food (Genesee Scientific, Cat #: 66–113) in a 25°C incubator with controlled 12 h:12 h light:dark cycle. Two hundred flies were collected every 4 days from amplification stocks using CO_2_-anesthesia and allocated into groups of 20 per food vial to control for the effects of overcrowding stressors. Aging flies were kept at 25°C and flipped every 3–4 days into fresh food containing vials until they reached the desired age. For experimentation, the desired age of young and aged cohorts was 4–8 days-old and 28–32 days-old, respectively. The experiment was carried over four weeks and the overall experimental set up is shown in Figure 1. Respiration in young flies was measured during weeks 1 and 4 of the experiment while respiration measurement in older flies was conducted during weeks 2 and 3 of the experiment. During each week of measurement, 3 groups of 2 vials with 20 flies each from either the young or aged cohort were randomly selected (a total of ~120 flies out of the 200 originally collected). The remaining flies were discarded. Subsequently, 20 out of 40 flies from each of the 3 groups of 2 vials were subjected to one of the three treatment protocols, resulting in 20 Non-injected (Ni), 20 Tris-injected (Tris) and 20 FHV-injected (FHV) flies. Anesthetized flies were sequentially placed in randomly labeled vials (1–20) for the corresponding treatment (FHV, Tris or Ni). Numbered treatment labels were subsequently replaced with a random vial identification number (e.g., v1 - v60) (Figure 1A and Supplementary Table 1). This numbering process was utilized to blind the authors to fly treatment during the process of the experiment. The treatment corresponding to each sample was only revealed after oxygen consumption data collection was complete.

On the day of each respirometry experiment, prior to transport to the UAB Comparative Organismal Energetics Core (COEC), each fly was assessed for survival. If a fly died at a timepoint, the unique ID number on their vial was recorded. Vials containing treated flies were then transported in an isothermal container from the *Drosophila* laboratory to the COEC. During each respirometry measurement at the COEC, 20 flies were measured in the Loligo Systems Microplate. If a fly were to die prior to the experiment, the body was still placed in the corresponding wells. The only empty wells on the plate were negative controls. The flies were returned to the *Drosophila* laboratory and placed back in the 25°C incubator. This survival and respirometry measurement cycle was completed three times in order to assess the oxygen consumption of 60 individual flies per measurement day (Figure 1B).

To account for possible confounding variables while measuring oxygen consumption in the Loligo Systems Microplate, two additional randomization procedures were utilized to account for *Drosophila* circadian rhythm and well-specific measurement variation. First, a random number generator was used to assign each of the 60 flies to one of the three 24-well plates, (Figure 1C and Supplementary Table 1) which resulted in 20 flies of different treatments being exclusively measured between 9 am-11am (ZT2-4, Plate 1), 11 am–1 pm (ZT4-6, Plate 2), and 1 pm–3 pm (ZT6-8, Plate 3) Central Standard US Time (Figure 1D). The respiration for three “plate groups” was measured concurrently at the same time throughout the week, such that each fly was always measured at the same period of circadian rhythm. Furthermore, this randomization limits confounding of specific treatments with potential changes in respiration associated with circadian rhythm.

In addition to a weekly plate assignment, daily well assignment within the 24-well plate was determined using another random number generator. After being assigned to a plate on the first day of respirometry, 20 individual flies that received treatment plus four “blank” (empty) wells were then assigned to a randomized well position on the 24-well microplate for each subsequent measurement timepoint (e.g., 24 h, 48 h, and 72 h post-treatment) (Figure 1D and Supplementary Table 1). The blank wells acted as negative controls for oxygen leakage. Since this experiment depends on the analysis of changes in respiration of individual flies, the randomization of the treatment groups should limit well-specific variations in oxygen leakage or oxygen sensitivity.

### Virus stock and injections

FHV stock was a kind gift from Dr. Annette Schneemann (Scripps Research Institute, La Jolla, CA, USA). Virus solution was stored at −80°C. All injections were done using a nano-injector (Nanoject II, Drummond Scientific), as previously described [19]. The nano-injector was prepared by mounting a mineral oil-filled glass capillary (Cat. No 3-000-203-G/X, Drummond Scientific), and drawing up Tris or FHV solution. Flies in FHV-injection cohort were individually injected with 4.6 nL of 2.9 × 10^6^ 50% Tissue Culture Infective Dose (TCID50)/mL of FHV. Flies in the Tris-injection cohort were individually injected with 4.6 nL of 10 mM Tris-HCl pH 7.5 to control for the effect of the injection injury. Flies undergoing injection protocol were anesthetized in their vial using CO_2_, kept under CO_2_ anesthesia on a fly pad (Genesee Scientific), and positioned on their side using a paintbrush. The glass capillary was inserted into the thorax of the fly, under the wing, and a foot pedal connected to the nano-injector was pressed to inject 4.6 nL of solution. The fly pad, nano-injector, mineral oil, capillaries and paintbrush underwent 280 mJ of ultraviolet radiation over 12 minutes prior to and after each injection within a tissue culture hood. The Tris treatment cohort was injected prior to the FHV cohort as an additional precaution against cross-contamination. Each injected fly was then placed into its own food vial and allowed to rest at room temperature for 1 hour after injection. Flies in non-injection cohorts were anesthetized, placed into individual food vials and allowed to recover for 1 hour following anesthesia. None of the treatment cohorts were subjected to CO_2_ anesthesia for a period exceeding 5 minutes. It is worth noting that Tris and FHV-injected cohorts experienced longer exposure to anesthesia because they must be anesthetized to administer the treatments.

### Microplate respirometry

OCRs of individual flies were measured by personnel at the COEC using the Loligo Systems complete Microplate System. Each round of these experiments was designed to measure the respiration of 60 individuals (20 Ni, 20 Tris, 20 FHV) over the period of three days post-treatment. Inside the 24-well Microplate, each of the 20 flies were placed inside one airtight 80 μl glass well, which contains an oxygen sensor. The sensor detects the oxygen concentration within the chamber and records oxygen levels at specified time intervals for the duration of the experimental measurement period. The oxygen unit reported by the Microplate System is mmol/L. This allows the calculation of the oxygen consumption rate. Four “blank” wells were left empty in each measurement and served as negative controls for normalization. Thus, the difference between the change in oxygen concentration between the blank wells and the well containing *Drosophila* could be attributed to the physiological activity of the organism.

Upon arrival at the COEC facility, flies were anesthetized using CO_2_ and placed into their corresponding well according to the sample randomization protocol (Figure 1D and Supplementary Table 1). Once the flies had been positioned in their respective wells, the microplate was sealed, and oxygen concentration was measured in each well for between 60 and 75 minutes. The first 15 minutes of oxygen measurement were excluded from analysis to allow for both animal acclimation and system stabilization after manipulating the flies. Thereafter, the oxygen concentration during the period of 15–59 minutes was measured and used for subsequent analysis.

### Survival of FHV infection assay

In parallel of the respirometry measurements, a survival assay was conducted at 25°C to determine fly mortality following FHV infection. Separate cohorts from the ones used for respirometry of *Oregon-R* male flies were aged to 4–8 d and 28–32 d, and subsequently individually injected with Tris or FHV using the method described above. After injection, triplicates with *n* = 10 flies of the same treatment were placed in food vials, allowed to recover for 1 hour at room temperature, and placed in the 25°C incubator with controlled 12 h:12 h light:dark cycle. Their survival was recorded every 24 hours.

### Statistical analysis

Power analysis for the present study was done in G*Power (version 3.1.9.7). With 240 animals across the three treatments and three measurements, we have 80% power to detect effect sizes as small as Cohen’s f(V) = 0.23 from a Repeated Measures ANOVA for within-between interaction.

Randomization procedures were implemented using the RAND function in Microsoft Excel (version 16.62, see Supplementary Table 1). Statistical analyses were performed using SAS software (v. 9.4 for Windows, SAS Institute, Cary NC, USA). In the first step, we consolidated and organized the oxygen concentration curves measured during the 15–59-minute period of microplate respirometry. The mean OCR was calculated for each non-overlapping 5-minute window, and these means were used to calculate the mean OCR for the entire duration of the measurements for each individual. Finally, the mean OCR of the blanks on each plate was subtracted from the mean OCR for each individual. OCR values for individual datapoints are plotted in respective figure graphs. Although dead flies were loaded onto the plate for measurements, their OCRs were considered as ‘missing’ and excluded from any statistical analyses. The change in oxygen concentration over each non-overlapping 5-minute window was then evaluated using linear mixed model accounting for repeated measures (24 h, 48 h, and 72 h post-treatment) adjusting for ‘Treatment’ (No injection, Tris injection, and FHV injection), ‘Time Post-Treatment’ (24 h, 48 h, and 72 h), ‘Zeitgeber Time’ (ZT2-4, ZT4-6, and ZT6-8), ‘Age’ (young, aged), and ‘Well position’ (A1-D6). In addition to these main effects, we also included three two-way interactions (‘Age * Treatment’, ‘Treatment * Time Post-Treatment’, and ‘Age * Time Post-Treatment’) and one three-way interaction (‘Age * Treatment * Time Post-Treatment’). To assess how OCR differed among the variables of interest, least squares means (LS-means) for OCR were calculated for main or simple effects from the model. LS-means are model-based, estimated means of OCR within each level of the variable of interest, averaged over covariates and factors. When the mixed model indicated a significant association between a variable and OCR (type 3 F-test *p*-value < 0.05), pairwise post-hoc tests were performed to evaluate whether the LS-means of OCR significantly differed between the levels of the variable. Pairwise comparisons of the OCR LS-means were performed using the *t* test statistic to evaluate differences within levels of each variable. Although defining families of comparisons for multiple-comparison correction is not standard for large mixed models with multiple factors [45] as in the current study, we performed a Tukey-Kramer post-hoc test to correct for each “slice” within an interaction (e.g., ‘Treatment * Time post-treatment’).

Post-treatment mortality between treatments within post-treatment time was analyzed with separate Chi-square (χ^2^)- tests, and by a logistic regression analysis.

Using GraphPad Prism (v. 9.5.0), a log-rank statistical test that compares Kaplan-Meier curves was employed to determine differences in survival duration between age and treatment groups at 25°C in the respirometry study and the survival experiment that we conducted in parallel. The survival probabilities of aged and young flies following Tris- and FHV-injections at different time points (24 h, 48 h, and 72 h post-treatment) were compared. A log-rank test using the SAS software (v. 9.4 for Windows, SAS Institute, Cary NC, USA) was performed to compare survival of FHV infection and Tris treatment within the respirometry and the independent survival experiments. Flies that were not dead at the end of the study period were censored observations.

Lastly, we assessed differences in OCR within experimental variables (‘Age’, ‘Treatment’, ‘Zeitgeber Time’) and post-treatment mortality. Two separate ANCOVA models were estimated, both comparing OCR at 24 h post-treatment by experimental variables and included an indicator of future mortality. In the first ANCOVA, the mortality indicator was defined as alive vs. dead at 48 h, while in the second ANCOVA this was defined as dead at 48 h, alive at 48 h but dead at 72 h, and alive at both 48 h and 72 h. We compared OCR in pairwise comparisons for those effects that were significant in type 3 tests.

Throughout the statistical analysis, a *p*-value of < 0.05 was considered significant. Adjusted *p* values (*p* adj) for multiple comparisons are shown.

### Data availability

Raw data respirometry files and code used for the statistical analysis in SAS is available at https://osf.io/b395q/.

## Supplementary Materials

Supplementary Figure 1

Supplementary Table 1
