# Supplementary material for: RNA virus-mediated changes in organismal oxygen consumption rate in young and old Drosophila melanogaster males
Source: Aging (Albany NY). 2023 Mar 22;15(6):1748–67. doi: 10.18632/aging.204593 (PMC10085608; doi:10.18632/aging.204593)
Supplement: Supplementary Figure 1 [file aging-15-204593-s001.pdf]

SUPPLEMENTARY FIGURE

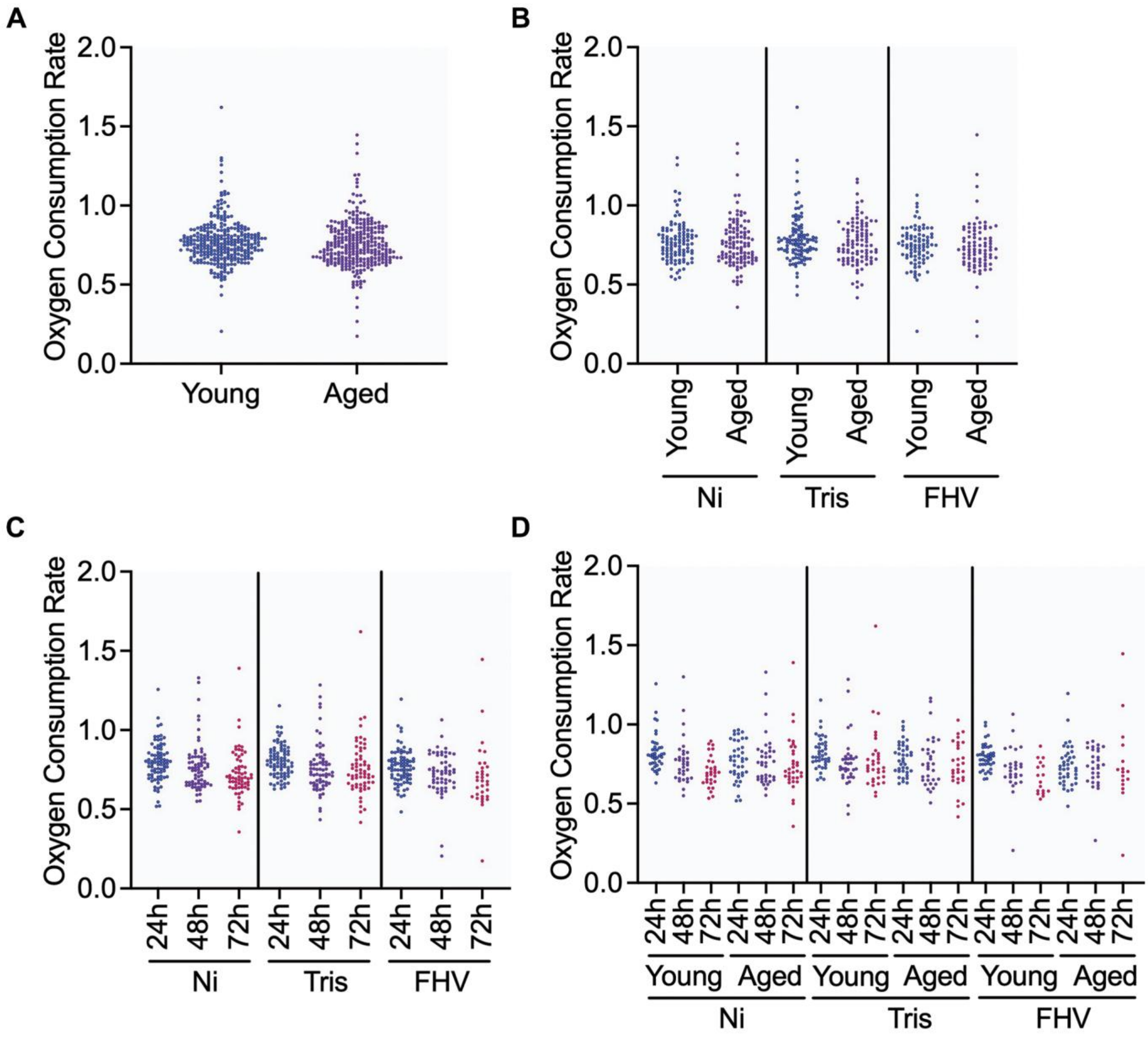

Supplementary Figure 1. Graphs showing pairwise comparisons of variables with non-significant main (**A**) or interaction (**B**–**D**) effect(s) on OCR: 'Age' (**A**), 'Treatment * Age' (**B**), 'Treatment * Time Post-Treatment' (**C**) and 'Treatment * Age * Time Post-Treatment' (**D**). OCR measurement values for individual datapoints are plotted. Each symbol represents an individual OCR measurement on a fly.
